# Platelet dysfunction in injured patients

**DOI:** 10.1186/s40591-014-0037-8

**Published:** 2014-12-19

**Authors:** Noelle N Saillant, Carrie A Sims

**Affiliations:** Division of Traumatology, Department of Surgery Critical Care and Acute Care Surgery, University of Pennsylvania, University of Pennsylvania, 3400 Spruce Street, 5 Maloney, Philadelphia, Pennsylvania USA

**Keywords:** Trauma, Hemostasis, Platelet activation, Platelet function, Platelet storage lesion, Platelet mitochondrial dysfunction, Coated platelets, Resuscitation, Coagulopathy

## Abstract

A renewed understanding of Trauma Induced Coagulopathy (TIC) has implicated platelets as a crucial mediator and potential therapeutic target in hemostasis. While the importance of abnormal coagulation tests is well described in trauma, there is a paucity of data regarding the role of platelets in coagulopathy. New coagulation models, namely the cell-based-model of hemostasis, have refocused attention toward the platelet and endothelium as key regulators of clot formation. Although platelet dysfunction has been associated with worse outcomes in trauma, the mechanisms which platelet dysfunction contributes to coagulopathy are poorly understood. The goal of this review article is to outline recent advances in understanding hemostasis and the ensuing cellular dysfunction that contributes to the exsanguination of a critically injured patient.

## Introduction

Hemorrhage continues to be a leading cause of early, potentially salvageable traumatic death. Mortality from hemorrhagic shock occurs quickly, with 80% of patients dying within the first 2 hours of hospital presentation [1]. The ability to form hemostatic clot is critical to the arrest of hemorrhage, yet in critically injured patients, hemostasis is severely compromised with over 25% of trauma patients presenting to the emergency room with a complex, biochemical coagulopathy known as trauma induced coagulopathy (TIC) [2-5]. Compared with patients who do not have coagulopathy, those with clotting dysfunction have a threefold to fourfold greater mortality [2,6,7].

The body’s ability to synthesize functional clot is critical to the arrest of hemorrhage, yet in critically injured patients, hemostasis is severely compromised.

The platelet, or thrombocyte, is the cellular agent responsible for clot formation. Despite functioning as the building block for hemostasis, the role of platelets in trauma-associated coagulopathy has been largely underappreciated. It is clear that platelet count and functional quality correlate with risk of death. Blood samples from trauma resuscitations show that even subtle disturbances in platelet quality and quantity can translate into a 10-fold increase in mortality [7-10].

Historically, TIC was first noted in military casualties when abnormalities were detected in routine lab studies [11]. Elevations in the international normalized ratio (INR), prothrombin time (PT) and the partial thromboplastic time (PTT) led the trauma community to focus their investigations on the protease- based reactions of the clotting cascade [11,12]. TIC was purported to occur in the setting of factor consumption and dilution from massive volume resuscitation. In an effort to minimize the hemodilution of clotting factors, resuscitation strategies changed from crystalloid volume replacement to transfusion with a more equalized ratio of packed red blood cells to plasma and platelets. The transfusion of platelets and factor rich plasma was correlated with improved survival [13-15].

As research honed in on transfusion protocols, two important developments suggested that TIC involved more complex mechanisms than simply hemodilution [16,17]. First, Brohi detected that coagulopathy was present in patients prior to fluid and blood product resuscitation.

In a retrospective review of 1088 trauma patients, 25% of patients had a clinically detectable coagulopathy on initial presentation and prior to fluid resuscitation [2]. Secondly, a new cell based coagulation model proposed by Hoffman in 2001 emphasized platelets as active regulators of thrombin activation [18-20].

## Review

### The cell based model of hemostasis

**Figure 1:**
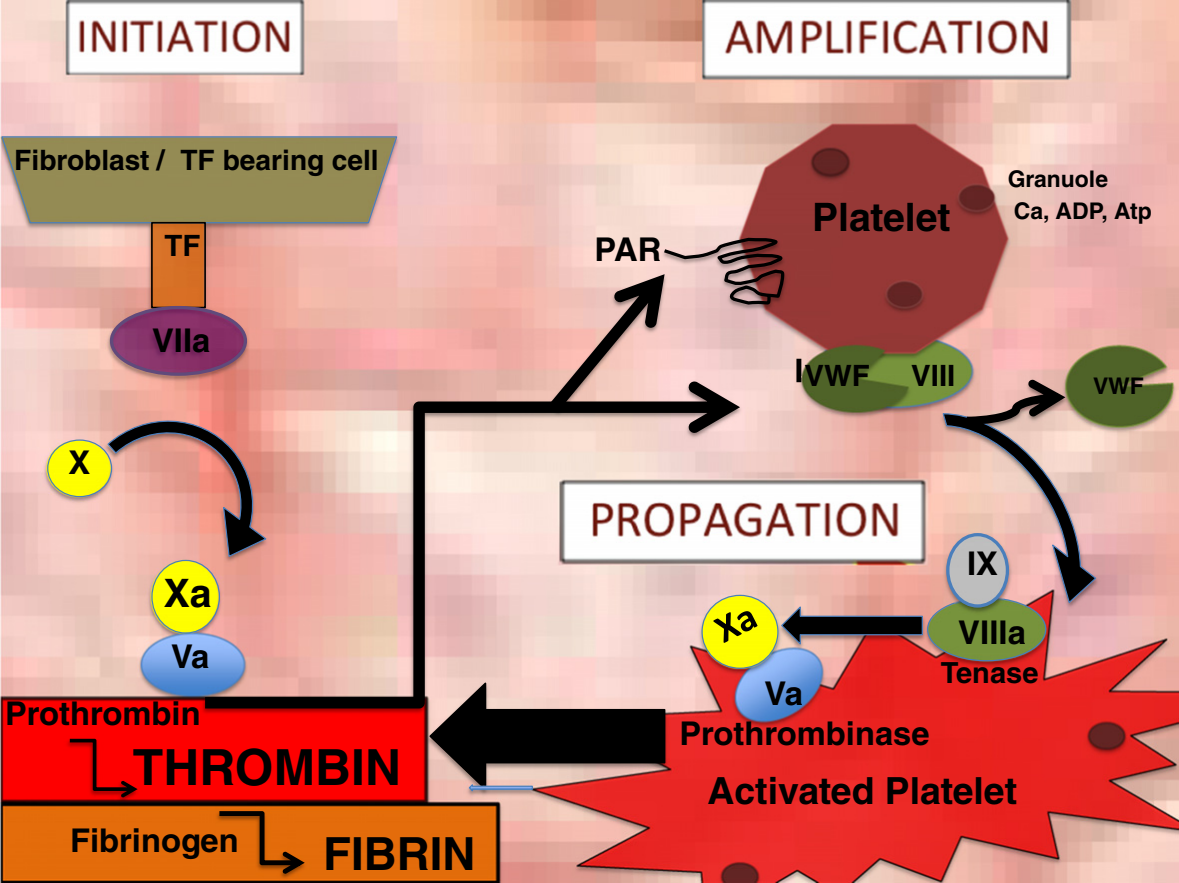
***Initiation:*** **Tissue Factor (TF) exposure binds factor VIIa.** The TF-VIIa complex activates factor Xa and factor Va leading to conversion of prothrombin to thombin. Thrombin generation activates platelets and initiates a positive feedback loop. *Amplification and Activation:* At the site of injury, platelets bind to exposed collagen and undergo a cytoskeletal transformation to the activated phenotype. Thrombin cleaves the VWF from the VWF/VIII complex thus activating factor VIII. *Propagation*: The tenase complex is formed by factor VIII complexing with factor IX. Factor IXa is then able to diffuse towards the platelet surface where it binds the surface bound, activated factor VIIIa. The prothrombinase complex is formed by the platelet membrane bound factor Va complexing with Xa formed from the tenase complex. The activated platelet membrane can then catalyze the Xa/Va prothrombinase complex to convert large amounts of prothombin to thrombin. Thrombin performs the final step in the deposition of fibrin clot by cleaving fibrinogen to fibrin [20].

Hemostasis in its simplest construct involves the production of a platelet plug and generation of thrombin and fibrin to produce a blood clot, or thrombus. In contrast to previous depictions of hemostasis, the cell-based model depicts thrombus formation as a series of three overlapping steps (initiation, amplification and propagation) rather than as a unidirectional proteolytic cascade (Figure 1).

#### Initiation

The first stage of the cell-based coagulation is termed “initiation”. The initiating signal for coagulation and platelet activation is tissue factor (TF). Tissue factor is an integral transmembrane glycoprotein that is variably expressed on the surface of certain cells including fibroblasts. The receptor shares homology with the interferon family of receptors, with a large extracellular portion that binds coagulation factor VIIa. Injury to a blood vessel wall exposes tissue factor to the plasma-based factor VIIa. The TF-VIIa complex utilizes proteolysis–dependent signaling to activate the coagulation factors Xa, and subsequently factor Va, leading to conversion of prothrombin to thombin. Fibrinogen, calcium, and activated coagulation factors (VIIa, Xa, Va , XIa, IXa) enhance thrombin generation via a positive feedback loop. The cell surface environment shelters thrombin from degradation by the inhibitors antithombin III and TFPI (Tissue Factor Pathway Inhibitor). Moreover, the thrombin that is retained at the cell surface plays an important function in platelet activation [20,21].

#### Amplification and activation

The scaffold of the prothrombinase complex centers around the platelet. At the site of injury, platelets bind to exposed collagen via a unique platelet glycoprotein, GIb/IX/V. The G1b component of this receptor binds Von Willebrand’s Factor (VWF) to tether the thrombocyte to the extracellular scaffold, while glycoprotein VI and α2β1 glycoprotein Ia-IIa reinforces this hold. By complexing thrombocytes to the collagen matrix, an adhesive platelet plug is formed at the site of injury that is capable of withstanding the shear stress of blood flow.

Although platelets are partially activated by binding to the extracellular matrix, full activation requires thrombin. Thrombin localizes to the platelet cell surface and fully activates platelets via its serine protease-activated receptors, PAR 1 and PAR 4. Protease–activated receptors (PARs) are G-protein coupled receptors that are activated by cleavage of their N terminus. In turn, PAR receptors activate platelets by augmenting inositol phosphate metabolism and intracellular calcium. Downstream G-protein signaling from PAR 1 and ADP activates Rho dependent phosphorylation of the myosin light chain and induces a cytoskeletal rearrangement in the activated platelet [21,22]. This leads to the formation of pseudopodia to increase surface contact with collagen and adjoining platelets.

Additional platelets are attracted to the site of injury and adhere to the platelet scaffold by their surface receptor, glycoprotein IIb/IIIa (αIIbβ3). The plug apparatus builds as activated thrombocytes release highly concentrated stores of factor V and other cofactors to form the growing clot. Thrombin cleaves the VWF from the VWF/VIII complex thus activating factor VIII at the platelet surface. The sequestration of both activated factor V and factor VIII at the platelet surface provides the necessary foundation for building the procoagulant complex to produce thrombin en-masse [20].

#### Propagation

Two complexes, the tenase complex and the prothrombinase complex, are formed at the platelet surface by activated factor VIII and factor V, respectively. The tenase complex is formed by factor VIII complexing with factor IX. Factor IX is activated by tissue factor bearing cells. Factor IXa is then able to diffuse towards the platelet surface where it binds the surface bound, activated factor VIIIa. The presence of the negatively charged platelet phospholipid surface environment enhances the activity of IXa/VIIa tenase complex. The tenase complex’s ability to form factor Xa is increased 1500 fold [23]. The tenase complex can therefore provide large amounts of factor Xa, to activate the second complex, known as the prothrombinase complex. The prothrombinase complex is formed by the platelet membrane bound factor Va complexing with Xa formed from the tenase complex. The activated platelet membrane can then catalyze the Xa/Va prothrombinase complex to convert large amounts of prothombin to thrombin. Thrombin performs the final step in the deposition of fibrin clot by cleaving fibrinogen to fibrin [20].

The prothrombinase reaction promotes a positive feedback loop. Increased thrombin stimulates further platelet activation and increased granule release. Fibrinogen and fibrin bind the integrin αIIbβ3 and promote platelet aggregation and clot stabilization [24]. This subsequently recruits more platelets and thus propagates new sites of platelet- prothrombinase activity.

The platelet-fibrin plug is stabilized by factor XIIIa which crosslinks fibrin and reinforces the clot with anti-fibrinolytic proteins. In particular, thrombin–activatable fibrinolysis inhibitor (TAFIa) is incorporated into matrix. TAFIa prevents clot degradation by decreasing the number of binding sites for endogenous plasminogen and tissue plasminogen activator. Finally, the intracellular portion of platelet integrin αIIbβ3 connects to the platelet’s intracellular actin and myosin network. Binding of fibrin to the extracellular portion of this receptor induces platelet contraction. The contractile force of the platelet, in turn, adds stress to the fibrin network, and subsequently causes clot retraction and stabilization [21,25].

In addition to supplying a phospholipid membrane structure for the coagulation complex to assemble, platelets actively secrete granules containing co-reagents necessary for clot propagation. The process of degranulation requires platelets to be activated. Secreted factors include calcium, ADP, ATP, serotonin, pyrophosphate, β thromboglobulin, and activated factor Va. These small molecules and proteins are critical cofactors for fibrin formation and platelet activation.

The cell-based model of hemostasis has implicated activated platelets as a crucial mediator of thrombus formation. Platelet activation, however, is a highly energetic process and and an emerging body of evidence has tied platelet activation to mitochondria and mitochondrial regulators of apoptosis [26,27]. Despite being anucleated cells, platelets retain caspases and other key mediators of apoptosis such as Bax, Bcl −2, and Calpain [26,28]. Moreover, the shrinkage, plasma membrane vesiculation, and bleb formation of apoptotic cells is reminiscent of the conformational changes observed in “activated” platelets. Furthermore, activated platelets increase their expression of P-selectin (CD62) and exteriorize phosphatidylserine (PS) on the platelet membrane: two events that are recognized as caspase-dependent markers of cell death in other cell lines [29]. While the significance of these mitochondrial-mediated events in activated platelets remains to be determined, the appearance of apoptotic features in stored banked platelets has significant clinical implications and correlates with decreased function in transfused platelets.

On the other hand, altered mitochondrial function in circulating platelets might *enhance* platelet function *in vivo*. An intriguing correlation has been established between the mitochondrial membrane potential (ΔΨ_m_) and the creation of a specialized subgroup of platelets, termed coated- platelets. Coated platelets are a select subpopulation of thrombocytes with escalated hemostatic potency that are induced *in vivo* by dual agonism with thrombin and collagen. Coated platelets use serotonin to retain procoagulant factors (e.g. factor V, fibrinogen, fibrin, VWF, fibronectin, α_2_-antiplasmin and thrombospondin) and phosphatidylserine on their surface [30]. As such, coated platelets ensure that the most potent site of clot formation is localized to the desired target [31,32]. This procoagulant profile is more likely to be induced in younger platelet populations and strongly supports prothombinase activity. The production of coated platelets appears to be linked to the loss of the mitochondrial membrane potential pore. Moreover the loss of ΔΨ_m_ serves as a stimulant for the creation of highly potent platelets, while the; inhibition of the mitochondrial permeability transition pore diminishes the numbers of coated platelets [33]. Although the physiologic consequence of these platelets is currently unknown, absence of these platelets leads to a bleeding diathesis in canine models [34] whereas an up-regulation of coated platelets may augment hemostasis. In clinical studies, diminished levels of coated platelets have been observed in subarachnoid hemorrhage and increased levels correlate with recurrent non-lacunar stroke and [35,36]. These divergent observations suggest that coated platelets are an important component of hemostasis. It is plausible that coated platelets may potentially be a therapeutic target for treatment of TIC.

## Assessing platelet function in trauma patients

Intuitively, hemostasis is likely dependent on a sufficient quantity of functional platelets. Observations across many patient cohorts demonstrate a correlation between the number of thrombocytes and the risk of death from hemorrhage. Higher platelets count upon arrival to the ICU has been associated with improved survival following abdominal aortic aneurysm rupture [37]. Similarly, a correlation between platelet count and survival has also been demonstrated in patients with traumatic injury. In a recent study of 389 severely injured patients, Brown and colleagues noted that for every 50 × 10^9^/L decrease in platelet number, the odds of dying increased by 17%. Importantly, this association held even when platelet levels were within the normal laboratory range of 100–450 × 10^9^/L [10]. While current transfusion guidelines recommend maintaining a platelet count of >50 × 10^9^/L this guideline may be insufficient in trauma patients and an incremental survival advantage is seen with platelets counts greater than 100 ×10^9^/L [10].

Although it is clear that an adequate number of platelets are important to survival, not all circulating platelets may be functional. Translational research studies have clearly demonstrated that critically injured patients develop physiologically significant platelet dysfunction, despite normal platelet counts. Using impedance aggrenometry to assess platelet function in 101 trauma patients, Kutcher et al., noted that platelet clotting was abnormal in an astounding 45% of patients on admission and in over 90% during their ICU course [8]. When platelet function was assessed using an agonist-impregnated cartridge that measures aperture occlusion by platelets, Jacoby et al. found that platelets were activated but functionally impaired. Notably, the most severely impaired platelet function was observed in brain-injured patients and in those who ultimately did not survive their injuries [38]. Interestingly, this study mirrored the results of others, implicating traumatic brain injury (TBI) as an independent risk factor for the development of coagulopathy [39-43].

The mechanisms for coagulopathy associated with TBI, however, have yet to be fully elucidated and may represent a unique coagulopathy, independent from TIC. One hypothesis is that the disruption of the protective blood brain barrier in TBI exposes an endothelium rich in tissue factor and platelet activating factor (PAF) [43,44]. Exposure to these two potent factors is hypothesized to activate platelets to a point of exhaustion and subsequent anergy [42,44]. The resultant platelet dysfunction limits thrombin production and stabilization of clot formation, thus compounding the effects of TIC in the polytrauma patient [45].

**Figure 2:**
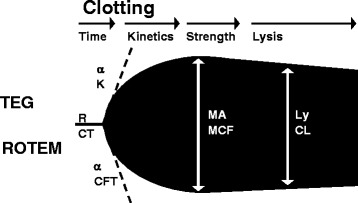
**Schematic TEG (upper half)/ ROTEM (lower half) trace with the corresponding measured variables shown.** Reaction time (R)/clotting time (CT), Clot formation time (K, CFT), alpha angle (), maximum amplitude (MA)/maximum clot firmness (MCF) and lysis (Ly)/clot lysis (CL). Reproduced with permission from Stissing, et al. [46].

The ability to test for platelet function in real time clinical situations remains technically complex. Recently, thromboelastography (TEG) and rotational thromboelastography (ROTEM) have re-emerged as potentially useful tools. TEG and ROTEM are viscoelastic assays that use a rotational force to evaluate the mechanical resistance of a clot over time. A computer analyzes the speed of clot initiation, the kinetics of thrombus formation, clot strength and lysis. The analysis is then graphically represented as a visoleastic plot. The contributions from clotting factors, fibrinogen and platelets can be subsequently inferred based on the morphology of the plot (Figure 2).

### TEG and ROTEM

The use of TEG in evaluating platelet dysfunction is an area of active investigation. Wolhauer et al., were one of the first groups to report abnormal platelet function in trauma patients using TEG-based mapping. Compared to healthy controls, trauma patients had an 86% inhibition of platelet response to ADP-agonism and a 44% inhibition of arachadonic acid agonism of platelet aggregation. Furthermore, in a subgroup of patients with traumatic brain injury, the level of ADP-inhibition distinguished survivors from non-survivors [39], The results of this study support the theory that platelet hypo-responsiveness may be a mediator of TIC.

Modified versions of TEG, for instance, the functional fibrinogen TEG (FF-TEG) and platelet mapping TEG (PM-TEG) have been developed to specifically differentiate the contribution of fibrinogen from that of platelets when evaluating a the coagulation profile. Using modified FF- TEG, investigators demonstrated that platelets, not fibrinogen, determine the clot strength throughout all time points of injury. Interestingly, FF-TEG levels predict outcomes; with every 1-mg/dL increase in FF-TEG levels leading to a 1% decrease in mortality [47]. Although not as well established, the PM-TEG eliminates the contribution of thrombin and fibrin to examine platelet receptor activity. The role of the PM-TEG in guiding blood product therapy has yet to be clinically validated [48].

### Platelet transfusions

Intuitively, low platelet counts and platelet dysfunction should be easily corrected by a platelet transfusion. However, platelets stored for transfusion, may not perform as well as native platelets. First described by Murphy et al. in 1971, the term “platelet storage lesion” (PSL) describes how banked platelets undergo a time-dependent degradation of function that mimics platelet activation and subsequent exhaustion [49-51]. The storage lesion is characterized by in-vitro abnormalities including derangements in metabolism [50,51] and reorganization of the cell structure. Stored platelets undergo a structural change and assume an activated phenotype by transforming from a discoid to a dendritic shape. Additionally, the mean platelet size decreases as platelets fragment into microvesicles. Glycoprotein expression is also altered with notable dysfunction of the GpIb-X-V receptor. As previously described, the ability of platelets to stabilize adhesion to the endothelial glycoprotein matrix is due, in large part, to the binding of glycoprotein1b of the GpIb-X-V receptor to Von Willebrand’s Factor. During storage, platelet-secreted metalloproteinases cleave the GpIbα and GpV portion of this receptor thereby reducing its adhesive potential. Furthermore, membrane clustering of the GpIb-X-V receptors mark transfused platelets for premature clearance from the bloodstream [52,53].

PSL also effects thrombus formation. Stored platelets exhibit alterations in calcium handling that subsequently impairs G -protein signaling as well as downstream phosphorylation of tyrosine kinases integral to TXA and ADP activation. As such, stored platelets exhibit a diminished response to ADP agonism and decreased aggregation. The diminished receptivity towards ADP may also be secondary to activation of the GTPase Rap 1. Rap 1 activates the platelet surface receptor GPIIb/IIIa during storage. Treating stored platelet with kinases that mediate Rap 1 activity restores resposiveness to ADP agonism, and reduces PSL-associated platelet activation [52]. Minimizing the effects of the PSL on platelet responsiveness is essential to ensure that transfused platelets remain functional.

A number of strategies have been investigated in order to prevent the development platelet storage lesion. In particular, platelet additive solutions have included a number agents thought to may inhibit platelet activation, including theophylline, prostaglandin E1, thrombin inhibitors, L-carnitine , citrate, adenosine, quinacrine, dipyramidole, ticlopidine, magnesium and potassium. Proteomics is currently being utilized to provide a comparative analysis of protein alterations in stored platelets. Developments from proteomics, such as the mechanism of Rap 1, have helped to elucidate the peptide-based changes associated with the PSL. There is hope that proteomics will continue to contribute to new investigational therapies [54].

The activation of blood-banked platelets is an energetically expensive process that is also linked with decreased mitochondrial potential [55]. Using high-resolution respirometry and fluorescence activated cell signaling, Villaroel et al. evaluated markers of mitochondrial function and apoptosis during the course of platelet storage. Interestingly, mitochondrial respiration and function deteriorated significantly within 2 days of storage. Reactive oxygen species and markers of apoptosis also significantly increased throughout storage. When the functional activity of platelets was tested by agonist stimulation, only 2% of the platelets responded appropriately at 72 hrs. These findings suggest that the integrity of mitochondrial respiration is compromised well before the intended five-day shelf life of stored platelets [56]. As such, additive solutions that support mitochondrial function may mitigate the platelet storage lesion and prolong the storage life of platelets.

## Conclusion

Platelets represent the next horizon in understanding coagulopathy and the opportunities to expand upon the current concepts are numerous. First and foremost, gaining a deeper understanding of the cellular mechanisms of platelet function will greatly enhance our model of cell-based hemostasis and trauma induced coagulopathy. Secondly, improvements in platelet function assays are needed to help direct individualized resuscitation and to identify patients at increased risk of dying from hemorrhage. Thirdly, mitochondrial bioenergetics appears to have a central role in platelet activation, platelet subspecialization, and in the development of the platelet storage lesion. As such, platelet mitochondrial function deserves further study in the quest to improve in-vivo platelet function and blood banking. Moreover,, the close association between platelet function and bioenergetics is intriguing and may provide a real time, clinically available surrogate marker of a patient’s mitochondrial profile [57]. The implications of studying platelets are far-reaching and important in trauma, and it is clear that we have much to accomplish.

## Abbreviations

TIC: Trauma induced coagulopathy
INR: International normalized ratio
PT: Prothrombin time
PTT: Partial thromboplastic time
TF: Tissue factor
TFPI: Tissue factor pathway inhibitor
VWF: Von Willebrand’s Factor
PAR: Protease activated receptor
ADP: Adenosine diphosphate
ATP: Adenosine triphosphate
TAFIa: Thrombin–activatable fribrinolysis inhibitor
PS: Phosphatidyserine
ΔΨ_m_: Mitochondrial membrane potential
MPTP: Mitochondrial permeabilitytransition pore
TBI: Traumatic brain injury
PAF: Platelet activating factor
TEG: Thromboeleastography
ROTEM: Rotational thromboelastography
(FF) TEG: Functional fibrinogen thromboeleastography
(PM) TEG: Platelet mapping TEG
COX: Cyclooxegenase
TXA2: Thromboxane A2
COX: Cyclooxegenase
TXA2: Thromboxane A2
PSL: Platelet storage lesion
PAS: Platelet additive solution
